# A novel computationally engineered collagenase reduces the force required for tooth extraction in an *ex-situ* porcine jaw model

**DOI:** 10.1186/s13036-023-00366-4

**Published:** 2023-07-17

**Authors:** Tamar Ansbacher, Ran Tohar, Adi Cohen, Orel Cohen, Shifra Levartovsky, Adi Arieli, Shlomo Matalon, Daniel Z. Bar, Maayan Gal, Evgeny Weinberg

**Affiliations:** 1 Department of Oral Biology, Goldschleger School of Dental Medicine, Faculty of Medicine, Tel Aviv University, 6997801 Tel Aviv, Israel; 2 Hadassah Academic College, 91010 Jerusalem, Israel; 3 Department of Oral Rehabilitation, Goldschleger School of Dental Medicine, Faculty of Medicine, Tel Aviv University, 6997801 Tel Aviv, Israel; 4 Department of Periodontology and Oral Implantology, Goldschleger School of Dental Medicine, Faculty of Medicine, Tel Aviv University, 6997801 Tel Aviv, Israel

**Keywords:** Collagen, Collagenase, Protein engineering, Tooth extraction, Minimally invasive medicine

## Abstract

The currently employed tooth extraction methods in dentistry involve mechanical disruption of the periodontal ligament fibers, leading to inevitable trauma to the bundle bone comprising the socket walls. In our previous work, we have shown that a recombinantly expressed truncated version of clostridial collagenase G (ColG) purified from *Escherichia coli* efficiently reduced the force needed for tooth extraction in an *ex-situ* porcine jaw model, when injected into the periodontal ligament. Considering that enhanced thermostability often leads to higher enzymatic activity and to set the basis for additional rounds of optimization, we used a computational protein design approach to generate an enzyme to be more thermostable while conserving the key catalytic residues. This process generated a novel collagenase (ColG-variant) harboring sixteen mutations compared to ColG, with a nearly 4℃ increase in melting temperature. Herein, we explored the potential of ColG-variant to further decrease the physical effort required for tooth delivery using our established *ex-situ* porcine jaw model. An average reduction of 11% was recorded in the force applied to extract roots of mandibular split first and second premolar teeth treated with ColG-variant, relative to those treated with ColG. Our results show for the first time the potential of engineering enzyme properties for dental medicine and further contribute to minimally invasive tooth extraction.

**Supplementary Information:**

The online version contains supplementary material available at 10.1186/s13036-023-00366-4.

## Introduction

Exodontia (i.e., tooth extraction) is among the most common clinical procedures in dentistry [1, 2]. The attachment of the tooth to the alveolar bone is primarily carried out with a group of collagen fibers known as periodontal ligament (PDL). Thus, regardless of the tooth extraction method, an important component required for safe tooth removal is careful disruption of the collagen fibers of the PDL, followed by the accurate delivery of the intact tooth [3, 4]. However, given the need for the application of additional surgical procedures such as root separation, reflection of a mucoperiosteal flap and removal of alveolar bone to gain access to the remnants of tooth roots, dental extraction potentially becomes a considerably invasive procedure [5, 6]. Such a procedure often creates damage to the surrounding soft and hard tissues, causing clinical complications [7–11]. Several breakthroughs in exodontia, such as periotomes, physics forceps, piezosurgery and various tooth extraction systems, have advanced safe tooth extraction. However, tools such as periotomes and piezosurgery implement the mechanical disruption of the PDL fibers while other appliances assisting in preserving bone socket dimensions by limiting the applied force to the vertical direction (e.g., the Benex® extraction system) [12–17]. Overall, these methods are based solely on the mechanical component, offering only a slight reduction in the amount of physical force required for tooth extraction.

Since enzymes can boost biochemical reaction rates [18], enzymatic degradation of the collagen fibers of the PDL prior to extraction per se could actually lead to a significant reduction in the physical force required for tooth delivery. Unlike mammalian collagenases that are part of matrix metalloproteinases (MMPs) [19–21] and usually cleave collagen at a single site, bacterial collagenases degrade collagen at multiple sites, turning collagen into short peptide fragments [22]. A variety of bacterial collagenases have been structurally and functionally characterized [23–30]. Among these, a truncated version of collagenase G of *Clostridium histolyticum* (ColG) was recombinantly expressed in *Escherichia coli* (*E. coli*), showing high expression yields [23, 31]. Indeed, several studies have analyzed and utilized enzymes or bacteria's ability to degrade collagen in the PDL, showing the feasibility of such biological-driven approaches [32–34]. Furthermore, native collagenase G extracted directly from *Clostridium histolyticum* is approved by the United States Food and Drug Administration (FDA) for treating Dupuytren’s contracture and Peyronie diseases, characterized by abnormal collagen deposition [35–37], further confirming the feasibility of enzymatically-driven degradation of the PDL collagen fibers *in-vivo*.

Various biotechnological applications require highly stable recombinant proteins that do not negatively affect enzymatic activity rates. However, natural enzymes often evolve with a tradeoff between stability and activity [38], which may not always be suitable for the desired application. This necessitates enzyme optimization to achieve a specific profile. Indeed, therapeutic enzymes could greatly benefit from a higher thermal stability profile, resulting in longer shelf life, lower aggregation rates, reduced immunogenicity, and improved activity in tissues. Moreover, although this correlation does not apply to all enzymes, in many instances, a thermally stable structure is associated with better function and higher yields of recombinant expression [39–44].

Enzyme durability with a longer half-life can be achieved through alternative approaches such as encapsulation or engineering of its structural features [45]. The latter can be accomplished using various computational approaches that have been developed to engineer protein variants with enhanced thermostability [46–51]. Although protein engineering is well established in a broad range of biotechnological and clinical applications [52, 53], its significance in dental medicine has not been demonstrated. Previous *in-silico* studies of collagenase focused on understanding the enzyme activity or analysis of the binding site, mainly for inhibiting the enzyme’s catalytic activity [54–57]. However, enhanced thermostability of ColG and the effect on collagen degradation were not explored. Inspired by the successful application of collagenase in medicine [58, 59], we have previously shown that injection of ColG into the PDL significantly reduces the force required to extract roots of split first and second mandibular premolar teeth in an *ex-situ* jaw model of 6-month-old domestic swine [60]. Herein, we harnessed the PROSS computational protein engineering techniques and seek for mutations in the primary sequence of ColG to further improve its thermostability and collagenolytic activity. Implementing the PROSS algorithm was shown to improve protein stability and heterologous expression levels for a variety of challenging enzymes and proteins [61, 62]. The PROSS web server assembles new backbone combinations, starting from a set of homologous yet structurally diverse enzyme structures, to optimize the amino acid sequence while conserving key catalytic residues [63]. Our results show that the novel enzyme resulting from the PROSS computational engineering (ColG-variant) reduces the force required for tooth extraction compared to ColG.

## Results

### Computational design of ColG-variant

Having an established assay for *ex-situ* evaluation of forces required for tooth extraction [60], we embarked on the engineering of a collagenase with enhanced thermostability. From the various alternative solutions of PROSS, we selected the least permissive one, which involved replacing 17 amino acids along the protein backbone. These replacements accounted for ~ 2.5% of the total active site protein length. This design ensures that the enzyme maintains its overall structure and activity. Indeed, in a benchmark study that evaluated multiple PROSS designs, the least permissive design exhibited significant improvement in expressions levels and thermal stability profile.

**Fig. 1:**
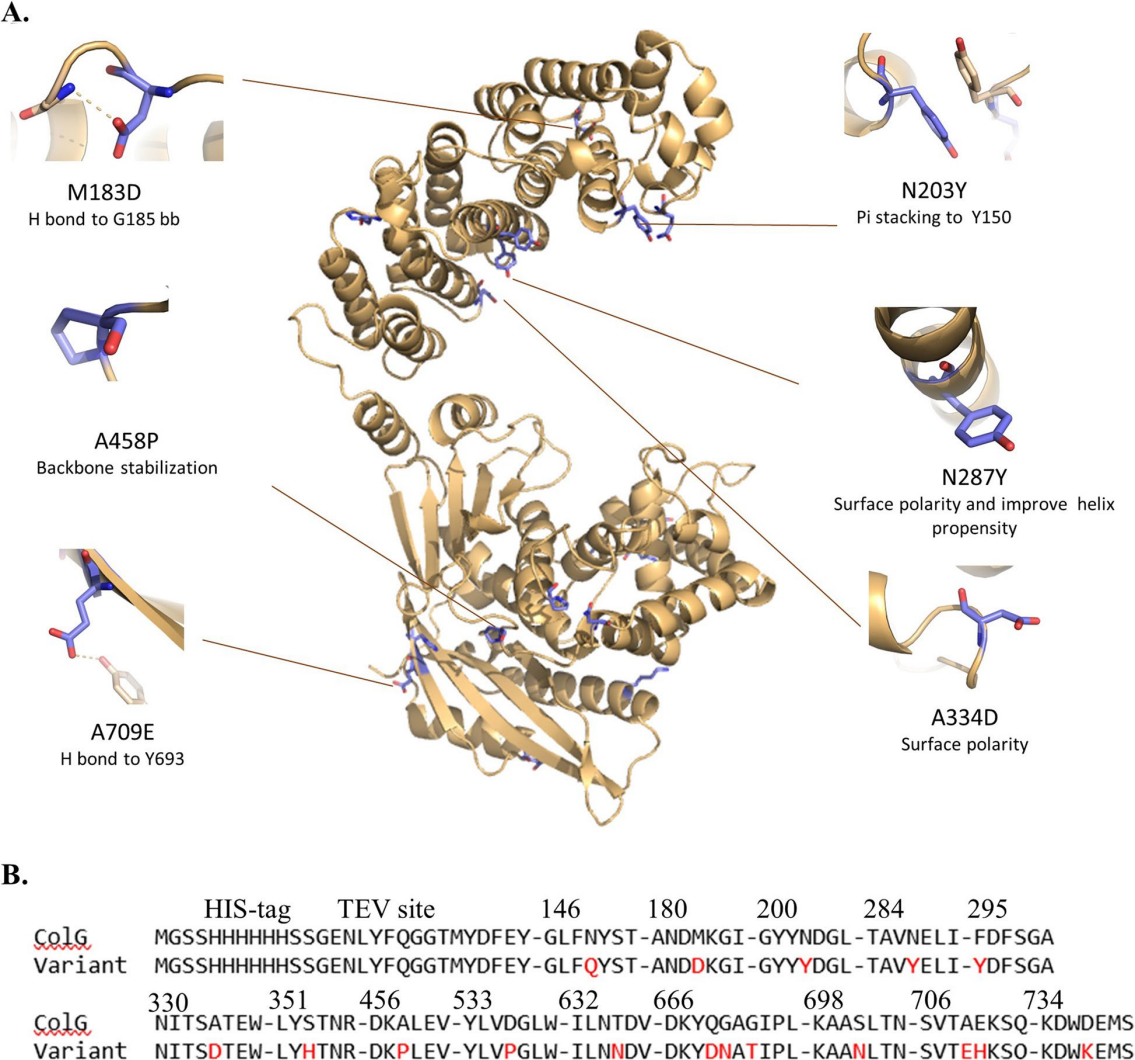
Illustration of ColG-variant. **A** The ColG structure is shown in brown, while mutations implemented in ColG-variant are depicted in purple (Selected mutations are shown). **B** Sequence alignment of ColG and ColG-variant in the specific regions, where mutations occurred (mutations in ColG-variant are denoted in red)

In addition to the N-terminus collagenase catalytic domain (Tyr119-Gly790), the overall structure of ColG is composed of a single polycystic kidney disease-like (PKD-like) domain, and various collagen-binding domains (CBD) [23]. The crystal structure of the N-terminus ColG reveals a saddle shape two-domain architecture. This latter shows full collagenolytic activity and is therefore selected as our template for further optimization. The catalytic domain comprises a highly conserved HEXXH Zn^+2^ binding motif as well as an Ca^+2^ binding site. Within this region, two conserved Gly, Gly493 and Gly494, the following edge strand—Leu495-Glu498 as well as Gln511-Phe515 are part of the collagenase substrate recognition site [29]. Figure 1A illustrates the position of the mutated amino acids on the ColG structure. As expected, most substitutions are positioned on the protein’s surface (purple residues in Fig. 1A), far from the active site of the enzyme. Moreover, the mutations do not interfere with the conserved Zn binding site residues or with the important collagen recognition site. This resulted in the replacement of surface-exposed hydrophobic groups into more hydrophilic residues. For example, F and G hydrophobic residues were mutated to Y/N/T hydrophilic residues in the F295Y, G670N and G672T positions. Figure 1B shows the sequence alignment of ColG and ColG-variant. In addition to hydrophobic to hydrophilic substitution, several new interactions contribute to enhanced stability, such as hydrogen bonding. For instance, Met 183 to Asp sets an H bond with Gly 185, and Ala 709 to Glu forms an H bond with the side chain of Tyr 693. The G672T mutation also permits the formation of a Hydrogen bond of the side chain of the Thr with the back bone of Ile 673. Although contribution of a single hydrogen bonding to stability is not substantial, such a network is essential for determining protein folding and structure. An additional interaction that plays a role in protein stability is the coulomb interaction, driven by the aromatic side chains [64]. The stacking interaction is formed via the substitution of Asn 203 Tyr, which constructs a pi–pi interaction with Tyr 150. Moreover, the replacement of Asn 287 with Tyr leads to a stabilizing of its alpha helix [65]. Additional stabilizing mutations of residues that are located within a flexible loop are the substitution of Ala 458 and Asp 536 with Pro, leading to a more rigid conformation of the protein [66].

### Evaluation of ColG-variant’s thermostability

**Fig. 2:**
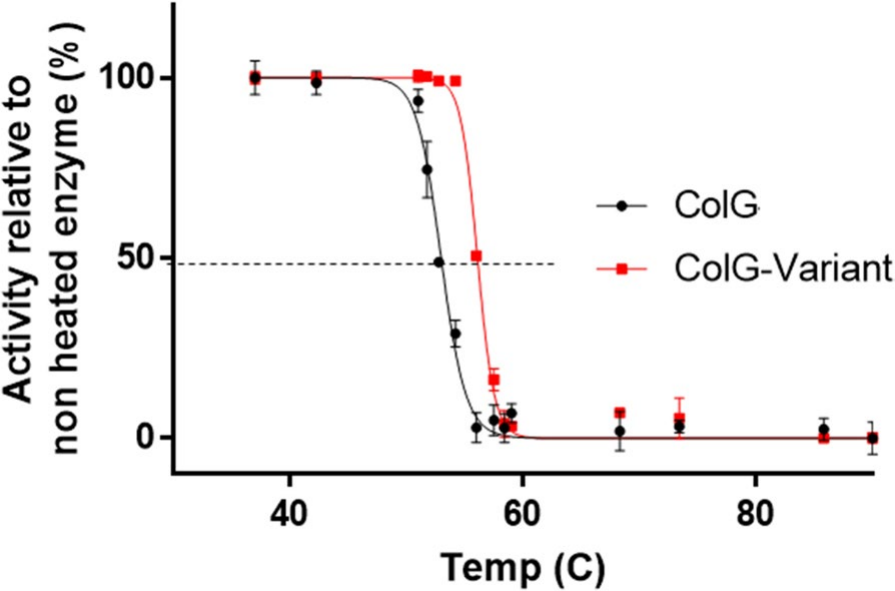
Tm Evaluation of ColG and ColG-variant. Enzymes were incubated at temperatures ranging from 30 °C to 90 °C for one hour and cooled down to 25 °C prior to the collagenolytic activity assay. Residual collagenolytic activity was plotted as a function of temperature for ColG (Black) and ColG-variant (Red). The data represent the mean of three replicates ± standard deviation

To evaluate the thermostability of ColG-variant, we examined its ability to digest native collagen following the incubation of the enzyme at variable temperatures, ranging from 30 °C to 90 °C. For this purpose, we relied on the biochemical collagenolytic activity assay of 3,4-DHPAA [31, 67]. This experimental approach enables evaluation of the activity of collagenase on a native full-length collagen, rather than on collagen-derived peptides. Figure 2 shows the relative residual collagenolytic activity of ColG and ColG-variant as a function of the temperature. Before the activity assay, the enzymes were incubated at variable temperatures for one hour and then cooled down to 25℃. The temperature at which the enzyme activity dropped to 50% relative to the activity of the non-heated enzyme is considered the melting temperature (Tm) of the enzyme. Thus, higher Tm suggests that the enzyme retained its activity at a higher temperature and is thus more thermostable. The Tm of ColG and the engineered ColG-variant were 52.9℃ and 56.6℃, respectively. This suggests the enhanced thermostability of ColG-variant.

### Measurement of force required for root extraction

**Fig. 3:**
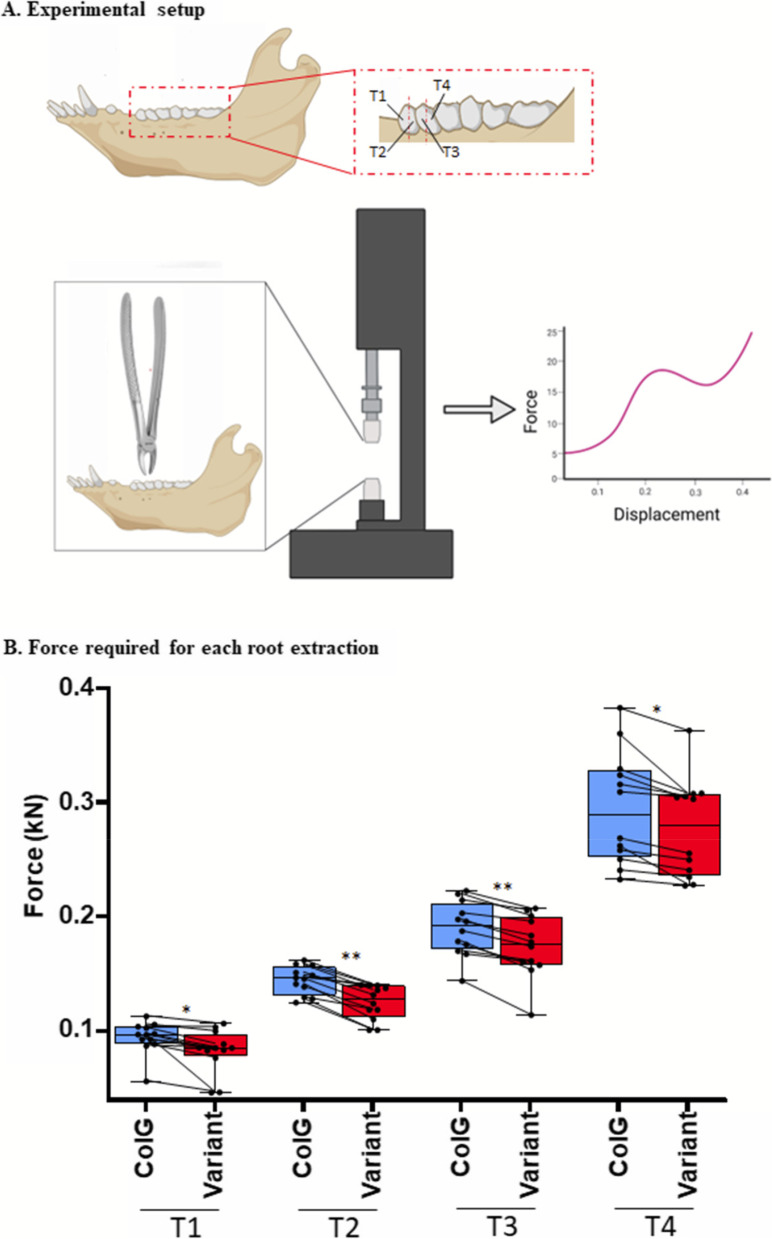
Measurement of tooth extraction forces in porcine jaw. **A** The first and second porcine mandibular premolar teeth were split to form four roots labeled as T1, T2, T3 and T4. Following 16 h from the injection of ColG or its variant, the extraction was performed using the tensile strength testing machine along the longitudinal axes of each extracted root, and the force vs. displacement was recorded. **B** Mean and dispersion of extraction forces of ColG (blue) and ColG-variant (red). Each contralateral pair of roots is marked by black circles and connective lines, and the horizontal line marks the mean force for each root. Statistically significant values are indicated above each paired data, *:*p* < 0.01, **:*p* < 0.001

To further evaluate the ability of the new enzyme to degrade collagen, we injected ColG-variant into the PDL of the split first and second mandibular premolar tooth roots (marked in Fig. 3A as T1 [mesial root of premolar 1], T2 [distal root of premolar 1], T3 [mesial root of premolar 2], and T4 [distal root of premolar 2]), in an *ex-situ* jaw model of 6-month-old domestic swine, as previously described [60]. ColG was injected into the corresponding roots on the contralateral side. Following incubation of 16 h, real-time recording of the pulling force vs. tooth displacement was performed using the tensile strength testing machine (Fig. 3A). Figure 3B shows the mean force (marked by a horizontal black line), as well as the values and dispersion of the root-specific maximal force applied to extract T1–4 in all jaws following treatment with ColG (blue) or ColG-variant (red). We found that the force required for extraction of each root was reduced with ColG-variant, by 12%, 13%, 8% and 6% for T1, T2, T3 and T4, respectively, with a total average reduction of 11%.

## Discussion

Protein engineering is a robust approach commonly used for imparting specific activity profiles to proteins for a broad range of applications. However, the use of bio enzymes in the field of dental medicine was rarely explored. Herein, relying on the ability of collagenase G to degrade PDL collagen fibers *ex-situ*, we aimed to engineer an enzyme with enhanced thermostability. The latter characteristic is often associated with improved activity or used as the starting point for further optimization of the enzyme. The engineering of new protein variants is commonly achieved by directed evolution methods. In a typical directed evolution experiment, random or semi-rational mutagenesis is used to generate a library of the target gene from which optimized variants are isolated following several screening and selection rounds [68, 69]. However, wet-lab methods generally require extensive experiments and are not efficient, especially for discovering optimized catalytic activity of enzymes. On the other hand, computational approaches have been shown to be effective in designing new proteins *de-novo* or based on the structure of a native protein as the starting point [70–73]. Here, we have used the PROSS algorithm to improve ColG stability. The algorithm has been successfully applied to various challenging enzymes and binding proteins, showing remarkable success in improving protein stability and expressibility, while maintaining wild-type activity levels [61]. Among the most dominant effects that govern protein folding and stability is the hydrophobic effect, whereby a-polar residues are buried within the stable protein structure, forming favorable Van Der Waals contact, while polar residues are presented at the protein surface [64]. Indeed, the point mutations in the ColG-variant (Fig. 1A) are located at the protein surface. Therefore, although every single mutation has a relatively low contribution to stability, the overall effect is not negligible.

The new ColG-variant was tested in two orthogonal assays. The first assay determined its thermostability by evaluating the ability of the enzyme to degrade collagen *in-vitro*, following incubation at varied temperatures. Enhanced thermostability is often correlated with improved characteristics that are important for an enzyme therapeutic application. In the context of enzymatically-driven exodontia, improved thermostability could mean accelerated degradation of the PDL collagen fibers due to better activity in the tissue and longer shelf life of the enzyme. We also noticed that albeit in a higher temperature, ColG-variant has a sharper activity decrease vs. temperature than its counterpart wild-type enzyme. This behavior is attributed to the enzyme’s net of new interactions, however, with no expected implications on clinical aspects. The second assay tested the superiority of ColG-variant in reducing the forces required for tooth extraction *ex-situ*, compared to ColG [60]. This may have a significant impact on the morbidity, implying fewer intra- and post-operative complications and reduced damage to soft and hard tissues surrounding the tooth being extracted. Furthermore, atraumatic or minimally invasive exodontia can facilitate the subsequent implant placement and restoration, thus shortening the overall time from procedure to final rehabilitation. Additional factors should be examined further, such as the reduction of the time period required from the moment of injection of the enzyme to extraction per se and the evaluation of minimal enzyme concentration that could lead to sufficient force reduction. Moreover, future research should evaluate the potency of ColG-variant in an *in-vivo* environment, additionally characterized by blood circulation and efficient regulatory immune system, which could affect the enzyme’s activity as well as its half-life in the tissue. Ultimately, considering the existing clinical applications of collagenase G, purified directly from *Clostridium histolyticum*, in orthopedics (i.e., Dupuytern’s contracture) [74, 75] and urology (i.e., Peyronie disease) [76], dental application of ColG-variant, characterized by enhanced thermostability, should be feasible.

## Conclusions

The research shows for the first time the application of engineered proteins in dental medicine. The engineering of an improved thermal-stable collagenase further reduces the force required for tooth extraction. It is concluded that the application of engineered biomolecules to impart a desired activity profile can advance non-invasive dental medicine.

## Methods

### Computational enzyme design

The initial structure of ColG is based on collagenase G from *Clostridium histolyticum* (PDB 4ARE) [25]. The catalytic domain of ColG comprises Tyr 119—Ala 790 [23], while the catalytic pocket itself ranges from Asp 398 to Gly 790 [29]. Zn^2+^ and Ca^2+^ ions are located at the catalytic domain and are essential for the enzymatic catalytic reaction [25]. To generate a more thermally stable variant, we initially relied on PDB structure 4ARE, maintaining its catalytic domain and the Zn^2+^ ion. However, the structure lacks the active Ca^2+^ ion. Therefore, as a preliminary preparation step, we replaced a water molecule within the enzyme cavity, with a Ca^2+^ ion. The latter was based on the well-resolved structure of collagenase H (ColH), PDB 4ARF. The modified structure was inserted into the PROSS web server (https://pross.weizmann.ac.il/step/pross-terms/). The PROSS web server assembles new backbone combinations, starting from a set of homologous yet structurally diverse enzyme structures, to optimize the amino acid sequence while conserving key catalytic residues [63]. PROSS uses phylogenetic analysis in combination with Rosetta atomistic design calculation in order to generate a set of mutations that are assumed to improve protein stability. PROSS conserves the essential amino acid sequence and side-chain conformations at the active site, yet, applies stabilizing mutations that are not rare among other homologs [61]. The two ions and their surrounding residues, especially the highly conserved residues, important for ion binding,—Glu 498, His 523, Glu 524 and His 527, were held constant throughout the calculations.

### Protein expression and purification

Hans Brandstetter [23, 25] generously provided the gene of ColG containing residues Tyr119-Lys1118 with an N-terminus His-tag followed by a TEV cleavage site. The gene of the new enzyme variant originating from PROSS was synthesized and cloned into the same pET15b vector. The plasmid was transformed into competent *E.coli* BL21(DE3) on an ampicillin agar plate. Colonies were transferred to Luria Browth (LB) medium supplemented with ampicillin (100ug/ul), and at OD_600_ = 0.8, protein expression was induced with the addition of 1 mM IPTG. For protein purification, cells were centrifuged, resuspended in a lysis buffer (50 mM NaPi, 300 mM NaCl, 10 mM Imidazole, pH = 8) and sonicated so as to disrupt them. The soluble protein fraction was isolated by centrifugation at 10,000 g, and the supernatant was applied to a 5 ml Ni^2+^ HisTrap FF column (Cytiva, USA). Following extensive washing with buffer-1 (50 mM NaPi, 300 mM NaCl, 40 mM imidazole, pH = 8), buffer-2 (50 mM NaPi, 1 M NaCl, 10 mM imidazole, pH = 8) and buffer-3 (50 mM NaPi, 300 mM NaCl, 20% Glycerol, pH = 8), the protein was eluted with buffer-1 containing 300 mM Imidazole. The elution fraction was concentrated using an Amicon Ultra-15 (Merck, USA) concentration tube (30,000-MWCO), and the buffer was changed to PBS by dialysis.

### Thermal stability assay

Thermal stability was evaluated by incubating 80 µl of the purified enzymes in a concentration of 200 µg/ml in 50 mM TRIS 5 Mm CaCl_2_ pH = 7.5 for 30 min in a PCR thermal cycler (C1000 touch, Bio-Rad, Germany). Incubation was carried out in variable temperatures, ranging from 37℃ to 90℃. Residual activity was then measured and compared with the activity of the unheated enzyme. The biochemical assay was performed based on the previously described protocol [31, 67]. The purified enzyme was incubated with collagen-I, and aliquots were mixed with 50 mM Tris buffer (pH = 7.5), 5 mM CaCl_2_ at a total volume of 200 µl in a 96 well plate at 37 °C. Aliquots of 50 µl were then mixed with 50 µl of 0.75 mM 3,4-DHPAA, 50ul of 125 mM sodium borate (pH = 8.0) and 50 µl of 1.25 mM NaIO4; and incubated for 30 min at 37 °C. The fluorescence intensity of the reaction mixture was measured by a spectrofluorometer (BioTek, Winoosky, VT, USA). The excitation and emission maxima were 375 nm and 465 nm, respectively.

### Jaw preparation and injection of collagenase into the PDL

The whole mandibles of a 6-month-old (90–100 kg) domestic swine close to slaughter were obtained from a local abattoir (Marsel Brothers Company, Haifa, Israel). A specially designed jaw stabilization device was employed, as previously described [60] and illustrated in Fig. 3A. Either ColG or its variant was injected into the PDL of contralateral roots of a mandibular porcine split first and second premolar teeth with the Wand Single Tooth Anesthesia System (Milestone Scientific, New Jersey, USA), as previously described [60]. Briefly, the outer soft tissues adjacent to the teeth were removed. Then, the first and second mandibular premolar teeth (PM1 and PM2, respectively), which contain two divergent roots, were split into four different roots T1, T2, T3 and T4 (Fig. 3A) [60]. Standard cartridges containing the local anesthetic solution for dental injection were emptied of their content and filled with either ColG or ColG-variant at a concentration of 4ug/ul. The injection was performed using a needle of 30G 2.54 cm that was inserted into the PDL space and advanced apically until stopped by the resistance of the alveolar bone proper. The injection was repeated at four sites around each root, on the buccal, lingual, mesial, and distal aspects. A total of 0.3 ml of 4 μg/μl was injected. Concentration was selected based on previous studies and clinical practice for injection of wild-type collagenase G for Dupuytren’s disease [60, 77].

### Measurement of force required for root extraction

The extraction force was applied by a loading machine (Instron Series 6800; Instron Corp., Canton, MA, USA) using a load cell of 2 kilonewtons and a crosshead speed of 10 mm/minute, until the root was completely removed from the alveolar socket. The force was recorded at a rate of 10 Hz. Assessment of the tensile force and displacement during the root extraction process was achieved via the designated software (Instron Series IX; Instron Corp.).

### Statistical analysis

Statistical comparisons between the force of the different extracted roots were performed by a paired t-test with two-tail distribution with unequal variance. For statistical analysis, significance was set as * = 0.01 ≤ *p* < 0.05; ** = *p* < 0.01.

### Graphics

Figure 3 was created with BioRender.

## Supplementary Information

**Additional file 1.**

## Data Availability

All data generated or analyzed during this study are included in this published article and its Supplementary information files.

## Abbreviations

PDL: Periodontal ligament
ColG: Collagenase G
MMPs: Matrix Metalloproteinase
WT: Wild type
FDA: Food and Drug Administration
PDB: Protein Data Bank
LB: Luria Browth
