# Supplementary material for: A novel computationally engineered collagenase reduces the force required for tooth extraction in an ex-situ porcine jaw model
Source: J Biol Eng. 2023 Jul 17;17:47. doi: 10.1186/s13036-023-00366-4 (PMC10353231; doi:10.1186/s13036-023-00366-4)
Supplement: Supplementary file 1 — Additional file 1. [file 13036_2023_366_MOESM1_ESM.docx]

**A Novel Computationally Engineered Collagenase Reduces the Force Required for Tooth Extraction in an *Ex-situ* Porcine Jaw Model**

Tamar Ansbacher^1,2,#^, Ran Tohar^1,#^, Adi Cohen^1^, Orel Cohen^1^, Shifra Levartovsky^3^, Adi Arieli^3^, Shlomo Matalon^3^, Maayan Gal^1,¶^ and Evgeny Weinberg^1,4,¶^.

^1^Department of Oral Biology, Goldschleger School of Dental Medicine, Faculty of Medicine, Tel Aviv University, Tel Aviv, 6997801, Israel.

^2^Hadassah Academic College, Jerusalem, 91010, Israel.

^3^Department of Oral Rehabilitation, Goldschleger School of Dental Medicine, Faculty of Medicine, Tel Aviv University, Tel Aviv, 6997801, Israel.

^4^Department of Periodontology and Oral Implantology, Goldschleger School of Dental Medicine, Faculty of Medicine, Tel Aviv University, Tel Aviv, 6997801, Israel.

^#^ Equal first authors.

^¶^ Evgeny Weinberg and Maayan Gal contributed equally to this study.

**Corresponding Authors**

Maayan Gal

Email: [mayyanga@tauex.tau.ac.il](mailto:mayyanga@tauex.tau.ac.il)

Evgeny Weinberg

Email: [evgenywein@gmail.com](mailto:evgenywein@gmail.com)

**Sequence of ColG**

MGSSHHHHHHSSGENLYFQGGTMYDFEYLNGLSYTELTNLIKNIKWNQINGLFNYSTGSQKFFGDKNRVQAIINALQESGRTYTANDMKGIETFTEVLRAGFYLGYYNDGLSYLNDRNFQDKCIPAMIAIQKNPNFKLGTAVQDEVITSLGKLIGNASANAEVVNNCVPVLKQFRENLNQYAPDYVKGTAVNELIKGIEFDFSGAAYEKDVKTMPWYGKIDPFINELKALGLYGNITSATEWASDVGIYYLSKFGLYSTNRNDIVQSLEKAVDMYKYGKIAFVAMERITWDYDGIGSNGKKVDHDKFLDDAEKHYLPKTYTFDNGTFIIRAGDKVSEEKIKRLYWASREVKSQFHRVVGNDKALEVGNADDVLTMKIFNSPEEYKFNTNINGVSTDNGGLYIEPRGTFYTYERTPQQSIFSLEELFRHEYTHYLQARYLVDGLWGQGPFYEKNRLTWFDEGTAEFFAGSTRTSGVLPRKLILGYLAKDKVDHRYSLKKTLNSGYDDSDWMFYNYGFAVAHYLYEKDMPTFIKMNKAILNTDVKSYDEIIKKLSDDANKNTEYQNHIQELVDKYQGAGIPLVSDDYLKDHGYKKASEVYSEISKAASLTNTSVTAEKSQYFNTFTLRGTYTGETSKGEFKDWDEMSKKLDGTLESLAKNSWSGYKTLTAYFTNYRVTSDNKVQYDVVFHGVLTDNGDISNNKAPIAKVTGPSTGAVGRNIEFSGKDSKDEDGKIVSYDWDFGDGATSRGKNSVHAYKKAGTYNVTLKVTDDKGATATESFTIEIKNEDTTTPITKEMEPNDDIKEANGPIVEGVTVKGDLNGSDDADTFYFDVKEDGDVTIELPYSGSSNFTWLVYKEGDDQNHIASGIDKNNSKVGTFKATKGRHYVFIYKHDSASNISYSLNIKGLGNEKLKEKENNDSSDKATVIPNFNTTMQGSLLGDDSRDYYSFEVKEEGEVNIELDKKDEFGVTWTLHPESNINDRITYGQVDGNKVSNKVKLRPGKYYLLVYKYSGSGNYELRVNK

**Sequence of ColG-variant**

MGSSHHHHHHSSGENLYFQGGTMYDFEYLNGLSYTELTNLIKNIKWNQINGLFNYSTGSQKFFGDKNRVQAIINALQESGRTYTANDMKGIETFTEVLRAGFYLGYYNDGLSYLNDRNFQDKCIPAMIAIQKNPNFKLGTAVQDEVITSLGKLIGNASANAEVVNNCVPVLKQFRENLNQYAPDYVKGTAVNELIKGIEFDFSGAAYEKDVKTMPWYGKIDPFINELKALGLYGNITSATEWASDVGIYYLSKFGLYSTNRNDIVQSLEKAVDMYKYGKIAFVAMERITWDYDGIGSNGKKVDHDKFLDDAEKHYLPKTYTFDNGTFIIRAGDKVSEEKIKRLYWASREVKSQFHRVVGNDKALEVGNADDVLTMKIFNSPEEYKFNTNINGVSTDNGGLYIEPRGTFYTYERTPQQSIFSLEELFRHEYTHYLQARYLVDGLWGQGPFYEKNRLTWFDEGTAEFFAGSTRTSGVLPRKLILGYLAKDKVDHRYSLKKTLNSGYDDSDWMFYNYGFAVAHYLYEKDMPTFIKMNKAILNTDVKSYDEIIKKLSDDANKNTEYQNHIQELVDKYQGAGIPLVSDDYLKDHGYKKASEVYSEISKAASLTNTSVTAEKSQYFNTFTLRGTYTGETSKGEFKDWDEMSKKLDGTLESLAKNSWSGYKTLTAYFTNYRVTSDNKVQYDVVFHGVLTDNG
